# Peanut components measured by ISAC: comparison with ImmunoCap and clinical relevance in peanut allergic children

**DOI:** 10.1186/s12948-021-00153-w

**Published:** 2021-08-09

**Authors:** H. K. Brand, M. W. J. Schreurs, J. A. M. Emons, R. Gerth van Wijk, H. de Groot, N. J. T. Arends

**Affiliations:** 1 Department of Pediatric Pulmonology and Allergology, Emma Children’s Hospital, Amsterdam University Medical Centres, Meibergdreef 9, 1105 AZ Amsterdam, The Netherlands; 2 Department of Immunology, Laboratory Medical Immunology, Erasmus Medical Centre, Rotterdam, The Netherlands; 3 Department of Pediatric Pulmonology and Allergology, Erasmus MC–Sophia Children’s Hospital, University Medical Center, Rotterdam, The Netherlands; 4 Department of Internal Medicine, Section of Allergology and Clinical Immunology, Erasmus Medical Centre, Rotterdam, The Netherlands; 5 Department of Allergology, Reinier de Graaf Hospital, Delft, The Netherlands

**Keywords:** Peanut allergy, Component resolved diagnostics, Ara h 2, Ara h 6, Multiplex analysis, ISAC, Food challenge test, Children

## Abstract

**Background:**

Specific IgE (sIgE) against the peanut component Arachis hypogaea (Ara h) 2 has been shown to be the most important allergen to discriminate between peanut allergy and peanut tolerance. Several studies determined sIgE cut off values for Ara h 2, determined by singleplex measurements. However, cut off values for Ara h 2 from multiplex arrays are less well defined. The aim of this study was to evaluate the correlation between Ara h 2 sIgE determined by singleplex versus multiplex measurements and to assess the diagnostic value of the different peanut components included in Immuno Solid-phase Allergen Chip (ISAC) multiplex analysis in children with a suspected peanut allergy.

**Methods:**

In this retrospective study we analyzed Ara h 2 sIgE values with singleplex Fluorescence Enzyme Immunoassay (FEIA, ImmunoCap) and multiplex microarray (ISAC) measurements in 117 children with a suspected peanut allergy. Also, other peanut components measured by ISAC were analyzed. Double blinded placebo controlled oral food challenges were used as golden standard.

**Results:**

Among all studied peanut components FEIA Ara h 2 sIgE showed the highest area under the curve (AUC, 0.922), followed by ISAC Ara h 6 and Ara h 2 sIgE with AUCs of respectively 0.906 and 0.902. Best cut off values to diagnose peanut allergy were 4.40 kU/l for FEIA Ara h 2 sIgE and, 7.43 ISU and 8.13 ISU for respectively Ara h 2 and Ara h 6 sIgE in ISAC microarray. Ara h 2 sIgE determined in FEIA and ISAC showed a good correlation (r = 0.88; p < 0.01).

**Conclusion:**

Ara h 6 and Ara h 2 sIgE in multiplex ISAC are both good predictors of clinical peanut allergy in Dutch children, and their performance is comparable to the use of Ara h 2 in singleplex FEIA. The simultaneous measurement of different peanut components using ISAC is an advantage and clinically useful to detect peanut allergic children that are Ara h 2 negative but sensitized to other peanut proteins such as Ara h 6.

## Background

Peanut allergy is an important and increasing cause of food allergy in children, affecting approximately 0.4–2% of the pediatric population in the Western World [1–3]. Clinical symptoms range from mild symptoms with only oral itching or dermal symptoms to systemic life-threatening anaphylaxis. Underdiagnosing a potentially severe peanut allergy can result in life-threatening allergic reactions in the home situation. On the contrary, over-diagnosis may impair patient and caregivers’ quality of life due to anxiety and unnecessary diets, may result in dietary shortages, higher health care costs and may increase the risk of developing an allergy during unnecessary elimination [4, 5]. Therefore it is important to distinguish a primary peanut allergy from sensitization only or from a peanut allergy due to cross-reactivity with tree or grass pollen (secondary allergy) resulting in no or merely mild symptoms. The oral food challenge (OFC) is the golden standard to confirm food allergy. Although this is an expensive and time-consuming intervention with the risk of a severe allergic reaction, it will also assess severity of the allergic reaction and the threshold of allergen concentration that will provoke an allergic reaction [6, 7].

Both skin prick test and serum sIgE to whole peanut extract have a high sensitivity but poor specificity in identifying peanut allergic patients [8, 9]. The development of component-resolved diagnostics (CRD) has enabled a diagnostic approach of peanut allergy at protein (component) level [10, 11]. With the measurement of sIgE to the individual peanut proteins, sensitization to peanut major allergens and cross-reactive peanut allergens can be distinguished. Component resolved diagnostics may help clinicians in their diagnostic approach, for example to prevent unnecessary food challenges and to assess the risk of a clinically relevant peanut allergy [11, 12].

The seed storage proteins Ara h 1, 2, 3 and 6 show a high degree of thermal and digestive stability. Due to these properties they are associated with a primary peanut allergy, potentially resulting in systemic allergic reactions including the risk of anaphylaxis [13–15]. Ara h 2 has already been shown to be an important allergen to discriminate between peanut allergy and peanut tolerance [16, 17]. Sensitization to Ara h 8, a cross reactive pathogenesis related protein family 10 (PR-10) protein, homologous to the major birch pollen allergen Bet v 1, predominantly induces mild local oral symptoms due to the lack of stability of this protein family during heating and proteolysis [18–20]. Ara h 9 belongs to the nonspecific lipid transfer proteins (nsLTPs) of the prolamin superfamily, which was reported as specific for peanut allergy in the Mediterranean area [19, 21].

Next to the determination of sIgE to individual components, it is also possible to simultaneously determine sIgE to a large number of components by the use of biochip technology. The ImmunoCAP ISAC (Thermo Fisher, Uppsala, Sweden) is such a multiplex assay that enables a semi-quantitative measurement of more than 100 food and inhalation allergen components including the peanut components. The small amount of blood required for the analysis, as well as the simultaneous measurement of multiple allergen components that can help in identifying an individual risk profile makes this allergen array based approach useful for characterizing specific IgE profiles in children with multiple inhalation and food allergies [9, 22]. Several studies investigated Ara h 2 cut off values, determined by singleplex measurements, to diagnose children and adults with a peanut allergy more precisely [16, 17]. However, cut off values for Ara h 2 from multiplex arrays are less thoroughly studied and no comparison of such values has been made between the two assays [14, 23, 24].

The aim of this study was to evaluate the diagnostic value of the different peanut components in ISAC microarray in a Dutch pediatric population with a suspected peanut allergy and compare Ara h 2 sIgE levels obtained from singleplex FEIA with ISAC microarray. These serologic values were examined on their clinical relevance, using double blind peanut challenge tests as golden standard.

## Methods

Data were retrospectively evaluated from a cohort of atopic pediatric patients in which an ISAC was performed during the period from August 2011 to March 2017 in an academic pediatric allergology unit (Erasmus Medical Centre, Sophia’s Children’s Hospital Rotterdam, the Netherlands) and in a regional pediatric allergology unit (Reinier de Graaf Hospital, Delft, the Netherlands). For this study, the patients that were sensitized to whole peanut extract determined by FEIA and/or skin prick test and in which a peanut challenge was performed were selected from the ISAC cohort. Indications to perform a peanut challenge were sensitization without earlier ingestion or sensitization with a positive history in the past to evaluate either tolerance induction or threshold and severity of the allergic reaction.

Data were obtained retrospectively, all interventions were conducted as part of regular patient care and used strictly anonymously, according to the principles of the Declaration of Helsinki and the code of conduct for medical research approved by the hospital’s Medical Ethical Committee. Parents gave their written informed consent before starting the challenge tests.

### Serology

Serum was collected during diagnostic workup. We used the FEIA-based system Phadia 250 (Thermo Fisher Scientific, ImmunoDiagnostics, Uppsala, Sweden) to measure specific IgE antibodies (ImmunoCap© sIgE method) against whole-peanut extract (f13), and Ara h 2 peanut component (f423). Specific IgE to the individual peanut components Ara h 1, Ara h 2, Ara h 3, Ara h 6, Ara h 8 and Ara h 9, were semi-quantitatively measured by the ISAC112© IgE microarray system (Thermo Fisher Scientific, ImmunoDiagnostics, Uppsala, Sweden) [25]. We defined positive sensitization as sIgE ≥ 0.35 kU/l in the FEIA and ≥ 0.3 ISAC standardized units (ISU) in the ISAC microarray. Reference values for FEIA (kU/l) and ISAC (ISU) are shown in Table 1.

**Table 1:** Reference values sIgE FEIA (kU/L) and ISAC (ISU)

FEIA (kU/L)		ISAC (ISU)	
< 0.35	Negative	< 0.3	Negative
≥ 0.35 to < 0.70	Low, class 1	≥ 0.3 to < 1.0	Low
≥ 0.70 to < 17.5	Moderate/high, class 2–4	≥ 1 to < 15	Moderate/high
> 17.5	Very high, class 5–6	≥ 15	Very high

### Peanut challenges

Double blind placebo controlled peanut challenges with gingerbread as matrix were performed. Before 2014, in the Reinier de Graaf Hospital, these challenges were performed in a 6-step dosing scheme according to Vlieg-Boerstra and Flinterman et al. [26, 27] with increasing dosages every 20–30 min of 0.2, 0.4, 2, 11, 53, 250 and 325 mg peanut protein equivalent. At the Erasmus Medical Center, and also after 2014 in the Reinier de Graaf Hospital, 7-step peanut challenges were performed with increasing dosages of (1), 3, 10, 30, 100, 300, 1000 and 3000 mg protein equivalent with 20 min interval for the initial 4 steps and 30 min interval after step 4 and scored according to PRACTALL guidelines [28]. The challenge was considered positive when objective symptoms occurred or when increasing subjective symptoms occurred on at least three subsequent doses. Objective symptoms were defined as angioedema, urticaria, vomiting, diarrhea, rhinoconjunctivitis, stridor, coughing, wheezing, hoarseness, collapse, tachycardia, and hypotension. Subjective symptoms were defined as abdominal pain, nausea and/or cramps, oral allergy symptoms, itchy throat or sensation of throat swelling, difficulty in swallowing, and ‘other’ symptoms such as drowsiness and irritability.

### Statistical analysis

Data were analyzed using SPSS. Differences were considered statistically significant at a P value less than 0.05. The frequencies were compared using the χ^2^ and the Fisher exact test. Non-normally distributed quantitative variables were compared using the Mann Whitney U test.

To assess the sIgE performance of the different peanut components receiver operation characteristics (ROC) curves were constructed. (version 20.0; SPSS Inc, Chicago, IL). Sensitivity, specificity, positive and negative predictive values (PPV and NPV) were calculated for sIgE to Ara h 6 and Ara h 2 with cutoff values of 0.35 kU/l for FEIA and 0.3 ISU for ISAC. Optimal cut off values of Ara h 2 and Ara h 6 in ISAC and Ara h 2 in FEIA were determined using ROC curves by maximizing the PPV.

Pearson correlation and Kappa statistics were performed to assess the concordance between Ara h 2 sIgE values obtained in FEIA and ISAC assays.

## Results

Double blind placebo controlled peanut challenges were performed in 117 children in our cohort of 384 atopic pediatric patients in which ISAC analyses were performed between August 2011 and March 2017. Ninety-six patients (82%) were included in the regional pediatric allergology unit. Fifty-three patients (45%) were challenged according to the PRACTALL guidelines and 64 according to the 6-step dosing protocol. The median age of the children was 6.9 years (range 0.8–15.9 years) and there was a male predominance (73%). Eighty-three percent of the children was diagnosed with eczema at time of inclusion or in the past, 49% had a doctor’s diagnosis of asthma and 57% suffered from allergic rhinoconjunctivitis symptoms with sensitization for one or more inhalation allergens.

Sixty-six children (54%) showed clinical reactions during the peanut challenge and 51 children were tolerant to peanut. The clinical characteristics of the peanut allergic and peanut tolerant children are summarized in Table 2. No clinically relevant differences in demographic characteristics, prevalence of asthma, allergic rhinitis, and eczema were found between the two groups. The reason to avoid peanut was significantly different between the two groups, in the peanut allergic group more than half of children avoided peanut because of an earlier allergic reaction to peanut while in the peanut tolerant group more children avoided peanut because of other reasons, such as another food allergy or sensitization without earlier ingestion.

**Table 2:** Clinical data of food challenge confirmed peanut tolerant and peanut allergic children

	Peanut tolerant (N = 51)	Peanut allergic (N = 66)	P value
Age, years, median (range)	7.6 (1.1–15.9)	7.0 (0.8–14.2)	NS
Gender, male, n (%)	39 (76%)	46 (66%)	NS
Eczema (in past), n (%)	43 (84%)	54 (82%)	NS
Asthma, n (%)	22 (43%)	35 (53%)	NS
Allergic rhinitis, n (%)	25 (49%)	42 (64%)	NS
Other food allergy, n (%)	38 (75%)	47 (71%)	NS
Reason avoiding peanut			
Allergic reaction in history, n (%)	13 (25%)	36 (55%)	P < 0.05
Sensitization, n (%)	10 (20%)	15 (23%)	NS
Other food allergy, n (%)	13 (25%)	5 (8%)	p < 0.05
Eczema, n (%)	4 (8%)	3 (5%)	NS
Unknown, n (%)	7 (14%)	7 (11%)	NS
Sensitization to birch pollen, n (%)	31 (56%)	45 (68%)	NS

Specific IgE levels against whole peanut extract and Ara h 2 determined by singleplex FEIA are shown in Table 3. From respectively 7 and 4 children FEIA data of whole peanut extract and Ara h 2 sIgE were missing. Skin prick tests for peanut in the 7 patients that did not show sIgE against whole peanut extract were all positive. Of the peanut allergic children, 97% was sensitized to whole peanut extract compared to 76% of the peanut tolerant group (p < 0.001). Median sIgE level to whole peanut extract was significantly higher in the allergic group compared to the tolerant group (8.3 kU/l vs. 1.7 kU/l, p < 0.001).

**Table 3:** Peanut extract and allergen-specific IgE in challenge confirmed peanut allergic and peanut tolerant children

FEIA	Peanut tolerant (N = 45)		Peanut allergic (N = 65)		P value
	N (%)	Median (range)	N (%)	Median (range)	
Peanut extract	34 (76)	1.7 (0–76.50)	63 (97)	8.1 (2.79 to > 100)	< 0.01

	Peanut tolerant (N = 49)		Peanut allergic (N = 64)		P value
	N (%)	Median (range)	N (%)	Median (range)	
Ara h 2	13 (27)	0.09 (0–4.40)	59 (92)	3.05 (0.03–462)	< 0.01

ISAC	Peanut tolerant (N = 51)		Peanut allergic (N = 66)		P value
	N (%)	Median (range)	N (%)	Median (range)	
Ara h 1	5 (10)	0.00 (0–2.80)	32 (49)	0.00 (0–67.49)	< 0.01
Ara h 2	14 (27)	0.00 (0–7.43)	62 (94)	3.71 (0–180)	< 0.01
Ara h 3	3 (6)	0.00 (0–0.69)	18 (27)	0.00 (0–12.49)	< 0.01
Ara h 6	14 (27)	0.00 (0–8.13)	61 (92)	3.64 (0–102.39)	< 0.01
Ara h 8	21 (41)	0.00 (0–136.61)	25 (38)	0.00 (0–23.89)	NS
Ara h 9	4 (8)	0.00 (0–8.62)	4 (6)	0.00 (0–2.69)	NS
Storage proteins					
None	30 (59)		2 (3)		< 0.01
1	10 (20)		4 (6)		0.03
≥ 2	11 (22)		60 (91)		< 0.01

Values are presented as number of positive (%) and median (ranges). Chi square and Mann Whitney U tests were performed

Specific IgE to Ara h 2 was present in 59 peanut allergic children (92%) compared to 13 children in the tolerant group (27%). The median level of Ara h 2 sIgE in the peanut allergic group (3.05 kU/l, range 0.14–321 kU/l) was significantly higher compared to the tolerant group (0.09 kU/l, range 0.00–4.40 kU/l).

### Peanut components in ISAC

Specific IgE values against the different peanut allergen components included in ISAC in peanut allergic and peanut tolerant children are shown in Table 3.

#### Sensitization patterns of sIgE to peanut components in peanut allergic children (N = 66)

Ninety-seven percent of the 66 peanut allergic children were sensitized to at least one peanut storage protein. Sensitization to Ara h 2 and Ara h 6 was most frequent in this group, detected in respectively 94% and 92% of the peanut allergic children, followed by Ara h 1 and Ara h 8 in respectively 49% and 38%.

Eighty-seven percent of the peanut allergic patients showed sensitization for both Ara h 2 and Ara h 6. There were no peanut allergic children sensitized for storage proteins Ara h 1 and Ara h 3, or the nsLTP protein Ara h 9, without co-sensitization for Ara h 2 or Ara h 6. Four peanut allergic children were not sensitized to Ara h 2, two of them were sensitized to other Ara h 6, of which one mono sensitization to Ara h 6. The other two did not show any sensitization to peanut allergens on ISAC. One of these patients had a slightly elevated sIgE to whole peanut extract (0.61 kU/l), and developed abdominal pain, acute rhinoconjunctivitis and itching skin after cumulative 1544 mg peanut protein during the peanut challenge. The other patient, without sensitization to whole peanut extract showed oral allergy symptoms and sensation of swollen throat upon subsequent dosages and challenge was stopped after a cumulative dose of 84 mg peanut protein. Both patients did not have symptoms during the peanut challenge on the placebo day. Skin prick tests to whole peanut extract were positive in both patients.

#### Sensitization patterns of sIgE to peanut components in peanut tolerant children

In the peanut tolerant group, 67% of the children (N = 38) was sensitized to at least one of the peanut components on the ISAC microarray (Table 3). Sensitization to Ara h 8 was most frequent in this group (40%), followed by Ara h 2 and Ara h 6 (both 27%). Forty-one percent (n = 21) showed sensitization to at least one of the storage proteins or the nsLTP protein Ara h 9. There were 19 mono-sensitizations in the peanut tolerant group, most of them (68%) showed a mono sensitization to Ara h 8.

#### Specific IgE to peanut components in ISAC in peanut allergic versus peanut tolerant children

**Fig. 1:**
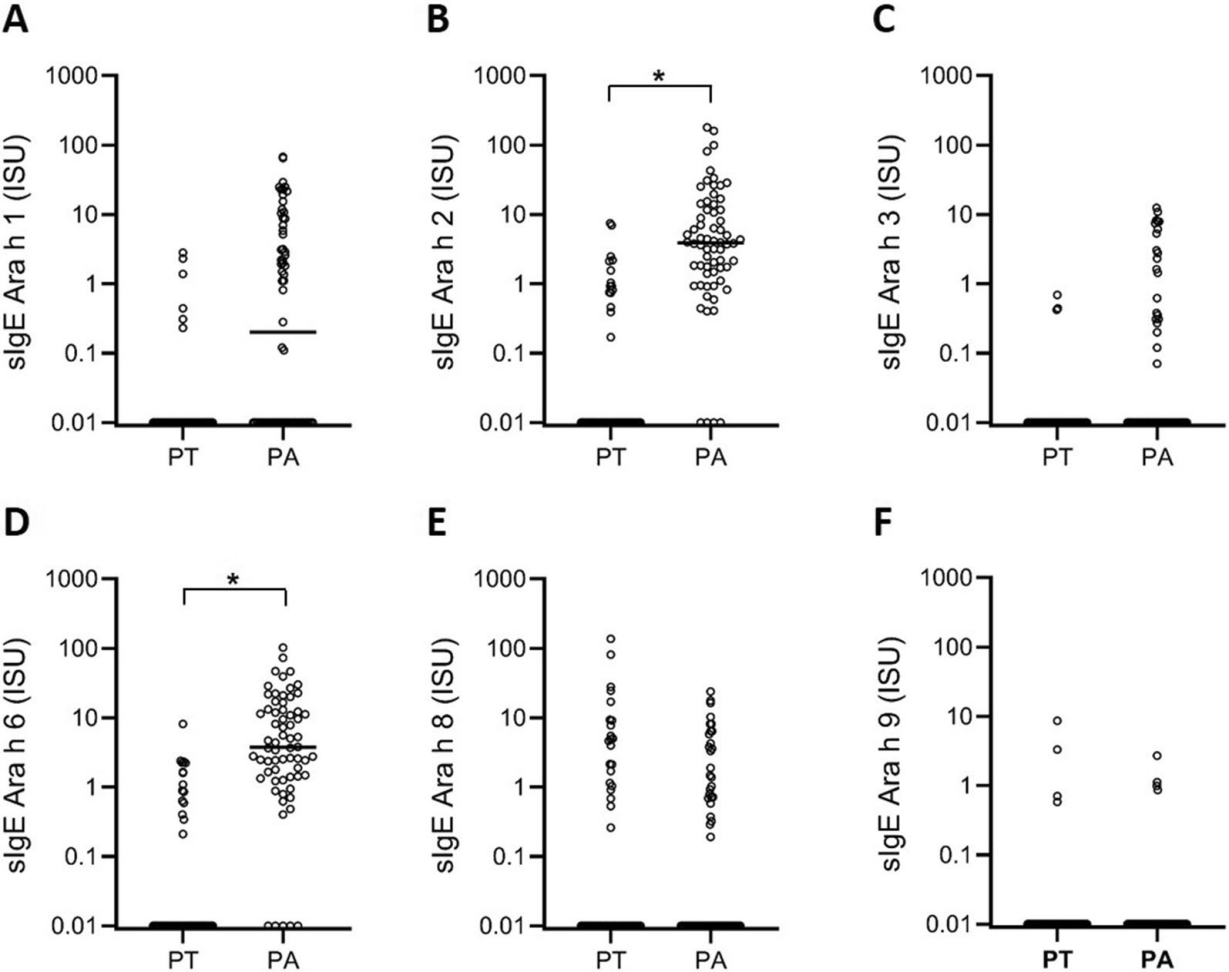
ISAC sIgE to peanut components in 66 challenge confirmed peanut allergic patients vs. 51 peanut tolerant patients. *PT* peanut tolerant, *PA* peanut allergic

Positive sIgE results for the peanut storage proteins Ara h 1, Ara h 2, Ara h 3, and Ara h 6 were more frequent in the peanut allergic group compared to the peanut tolerant group (Table 3 and Fig. 1). These differences were not observed for Ara h 8 and Ara h 9. In addition, the median levels of the different peanut storage proteins were significantly higher in children with positive peanut challenges compared to those with negative challenges (Table 4). Ara h 8 and Ara h 9 were not significantly different between both groups (p = 0.66 and p = 0.69 respectively). More multiple sensitizations were found in the peanut allergic group compared to the peanut tolerant group (95% versus 29%, p value < 0,001).

**Table 4:** Diagnostic performance of different cutoff points for FEIA and ISAC Ara h 2 sIgE, and ISAC Ara h Ara h 6 sIgE

Ara h 2	Sensitivity (%)	Specificity (%)	PPV (%)	NPV (%)
FEIA (kU/l)				
> 0.10	98	53	73	96
> 0.35	92	73	82	88
> 4.40	46	100	100	56
Ara h 2 ISAC (ISU)				
> 0.10	94	71	81	90
> 0.30	94	73	82	90
> 7.43	32	100	100	53
Ara h 6 ISAC (ISU)				
> 0.10	92	73	81	88
> 0.30	92	73	81	88
> 8.13	38	100	100	55

*PPV* positive predictive value, *NPV* negative predictive value

### Correlation between Ara h 2 sIgE in ISAC versus FEIA

A high degree of correlation was found between singleplex FEIA Ara h 2 and ISAC microarray Ara h 2 sIgE (r = 0.88, p < 0.01). At an individual level, 6 patients (5%) showed clinically relevant discrepancies. Two patients with a negative Ara h 2 sIgE on ISAC, showed low levels sIgE to Ara h 2 as determined by FEIA (0.53–0.70 kU/l). One of these patients developed itching mouth, abdominal pain and tiredness after cumulative 250 mg peanut protein during the food challenge This patient was sensitized to Ara h 6. The other patient was peanut tolerant.

Oppositely, 4 subjects with FEIA Ara h 2 sIgE levels below 0.35 kU/l (range 0.19–0.31 kU/l) did show a positive Ara h 2 sIgE as determined by ISAC (0.44–0.75 ISU). Two of them passed the peanut challenge, the other two were peanut allergic. One of these patients developed symptoms of wheezing, coughing and urticaria after cumulative 1440 mg peanut protein and was treated with adrenalin intramuscularly and salbutamol on the verum day. The other patient developed oral allergy symptoms and malaise after cumulative 325 mg peanut protein. Both patients experienced no symptoms during the placebo day. Both patients did also have low levels of sIgE against Ara h 6 as determined by ISAC and showed positive skin prick test to whole peanut extract.

### ROC curves

**Fig. 2:**
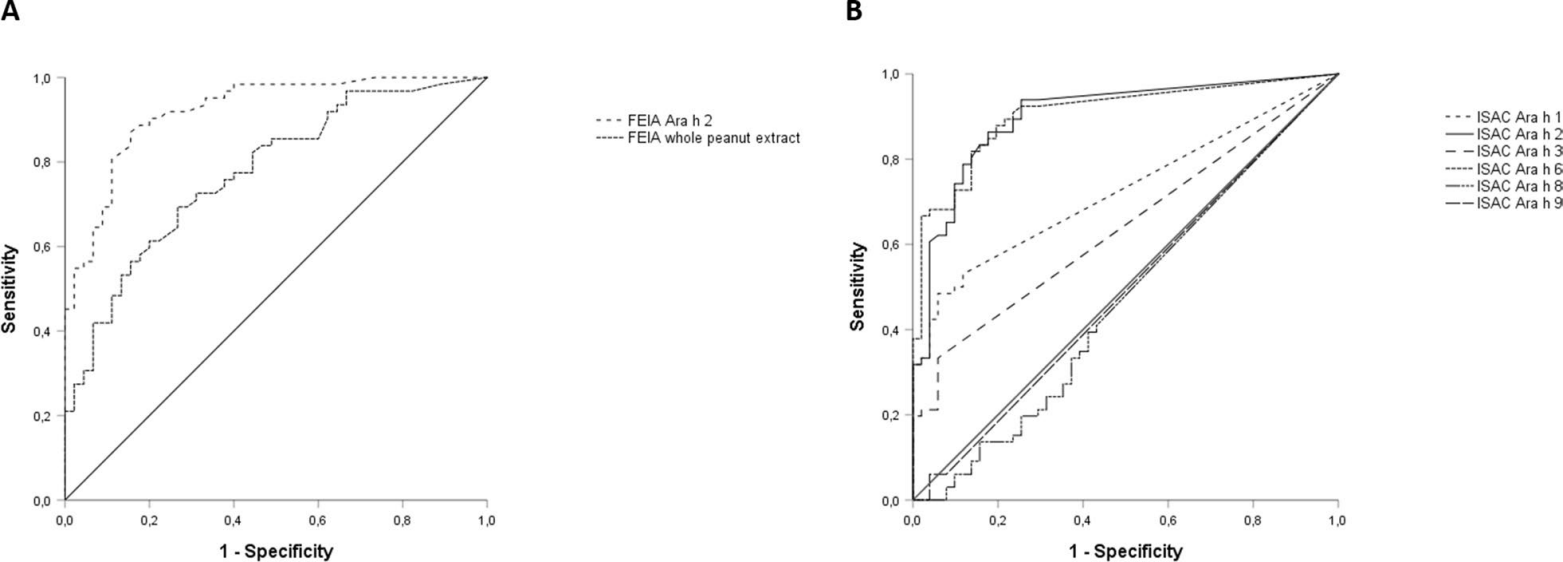
Receiver operating characteristic (ROC) curves for **A** FEIA sIgE to whole peanut extract and peanut component Ara h 2 and for **B** ISAC sIgE to the indicated individual peanut components

FEIA Ara h 2 sIgE showed the highest AUC (0.923), followed by ISAC Ara h 6 and Ara h 2 sIgE with AUCs of respectively 0.906 and 0.902 (see Fig. 2).

For FEIA Ara h 2 ≥ 0.35 kU/l, sensitivity for peanut allergy diagnosis was 92%, specificity 73% with a positive predictive value of 82%. Lowering the cut-off value to 0.1 kU/l for FEIA Ara h 2 sIgE improved sensitivity to 98% and the negative predictive value from 88 to 96%. A 100% positive predictive value was reached at a cut off value above 4.40 kU/l for FEIA Ara h 2 sIgE.

For both ISAC Ara h 2 and Ara h 6 sIgE the manufacturer’s cut-off value of < 0.3 ISU showed a sensitivity of 94% and 92% respectively. Specificity of both Ara h 2 and Ara h 6 was 73%. Lowering the cut-off to < 0.1 ISU did not improve the sensitivity and negative predictive value**.** Diagnostic accuracy was optimal at a cut off value of 0.815 and 0.665 ISU for respectively Ara h 2 and Ara h 6, with sensitivity and specificity of 86% and 82% for Ara h 2 and 88% and 78% for Ara h 6. PPVs reached 100% at cut off values above 7.43 ISU and 8.13 ISU for respectively Ara h 2 and Ara h 6 sIgE in ISAC microarray. Sensitivities, specificities, PPVs and NPVs for the different cut-off values are depicted in Table 4.

Using the upper cut-off value of 7.43 ISU and the lower cut off value of 0.3 ISU for Ara h 2 in ISAC analysis, the presence or absence of a peanut allergy could be predicted in 50% of the study population with 94% accuracy. By combining the cut-off values of Ara h 2 and Ara h 6 sIgE the absence or presence of peanut allergy can be predicted in 52% of the children with 97% accuracy.

## Discussion

In this Dutch pediatric population Ara h 2 and Ara h 6 sIgE determined using ISAC analysis are good predictors for peanut allergy and ISAC cut-off values are reliable and useful to predict a clinically relevant peanut allergy. Ara h 2 sIgE determined by multiplex ISAC microarray analysis shows a good correlation with Ara h 2 sIgE determined by singleplex FEIA measurement. To our knowledge, this is one of the first studies evaluating cut-off points of peanut components using multiplex ISAC analyses in peanut allergic and peanut tolerant children, diagnosed by double blind placebo controlled peanut challenge tests. The few other studies that have investigated sIgE cut-off values to diagnose a peanut allergy using microarray peanut components in children did not use food challenge tests as golden standard or performed peanut challenge tests in only a subgroup of the study population [14, 23, 24, 29, 30].

In our population Ara h 2 and Ara h 6 scored best in diagnosing peanut allergy. 100% PPV was reached at ISAC microarray cut off values of 7.43 ISU and 8.13 ISU for Ara h 2 and Ara h 6 sIgE respectively. Nineteen and 24 patients (16% and 21% of the study population) had an Ara h 2 and Ara h 6 value above this cut off value and could be classified as peanut allergic with 100% certainty. Klemans et al. calculated cut off values in diagnosing peanut allergy in adults using ISAC, and showed a 100% PPV at a cut off value of > 9.74 and > 2.40 for respectively Ara h 2 and Ara h 6 [31]. Looking at our population, 2 outliers increased the cut off values of Ara h 2 and Ara h 6 from 2.47 and 2.32 ISU to 7.43 and 8.13 ISU respectively. Both patients passed the 6-dose food challenge. However, in one patient information regarding home introduction was missing due to loss to follow-up and in the other patient, home introduction failed due to refusal by the child.

The few other studies that evaluated the performance of peanut components in diagnosing peanut allergy in children using ISAC analyses showed similar results revealing Ara h 2 and Ara h 6 as best performing allergens [14, 23, 24, 29, 30]. Although sensitization rates to the nsLTP protein Ara h 9 were higher in the Mediterranean studies, Ara h 2 and Ara h 6 still preformed best in predicting peanut allergy due to the high sensitization rate of Ara h 9 in the peanut tolerant children [14, 24, 29]. Cut off values in all studies vary most probably due to other study designs, study population and geographical location.

### Singleplex versus multiplex Ara h 2 analysis

The semi-quantitative sIgE measurements of multiplex microarrays are considered to be less sensitive for monitoring sensitization compared to singleplex measurements [32]. In our study, Ara h 2 sIgE values in singleplex FEIA and multiplex ISAC showed a strong correlation and we did not miss more peanut allergies using ISAC compared to FEIA Arah2 sIgE. In addition, the simultaneous measurement of other peanut components, Ara h 6 sIgE in specific, may improve the diagnostic performance of ISAC microarray. This is in line with other studies, which also showed comparable IgE recognition patterns and diagnostic sensitivities between multiple and singleplex determined peanut allergens [33–35].

### Cut-off values of singleplex Ara h 2

Our singleplex Ara h 2 sIgE data confirmed sIgE to Ara h 2 as a good predictor for peanut allergy in children [8, 16, 19, 36–45]. In our study we found a 100% positive predictive value for Ara h 2 sIgE determined by singleplex FEIA at 4.40 kU/l. Cut off values can vary due to study population and geographical location. To use cut off values in daily practice, clinicians has to determine and validate cut off values in their own specific population and region. Nevertheless, our results are quite similar to two other Dutch studies in comparable atopic pediatric study populations but in other regions of the country [36, 46].

In the study from Klemans et al. the negative predictive value of FEIA Ara h 2 sIgE improved to 100% accuracy lowering the lower cut-off value from 0.35 to 0.07 kU/l. They concluded that the need for peanut challenges could be reduced using Ara h 2 sIgE measurements. In our study population 5 patients (8%) of the Ara h 2 sIgE negative children, determined by FEIA developed objective allergic symptoms upon peanut challenge. This is in concordance with a recent systematic review based on 16 control studies in children showing that using the Ara h 2 sIgE cut-off value of 0.35 kU/l results in 8.1% false negative results [17]. Based on these findings we advise to be careful with peanut home introductions in Ara h 2 negative children due to the small risk of an allergic reaction at home.

In addition, peanut sensitization patterns can vary between different geographical regions and, other peanut components, such as the nsLTP protein Ara h 9 in Mediterranean regions, may be important in predicting systemic peanut allergic reactions. Although Ara h 2 is also an important marker of primary peanut allergy in Mediterranean regions [14, 24, 29, 47], sensitizations to Ara h 9 occur more frequently and more data are required to demonstrate the clinical performance of Ara h 9 in predicting systemic peanut allergic reactions in these region.

The small proportion of peanut allergic children with both very low or negative sIgE to Ara h2 and low whole peanut extract values but positive skin prick tests can be explained by either a lower threshold of sIgE to Ara h2 provoking allergic reactions or sensitization to other peanut minor components such as oleosins. Oleosins are lipophilic allergens that are underrepresented in whole peanut extracts because they are poorly soluble in aqueous solutions [48].

### Peanut component patterns

We evaluated sensitization patterns of different peanut components in peanut allergic and peanut tolerant children. Our data showed that Ara h 2 and Ara h 6 are the most common peanut allergens in Dutch children with a peanut allergy (94% and 92%), followed by Ara h 1 and Ara h 8 detected in 49% and 38%.

This is similar to the 90% prevalence of Ara h 2 sensitization in peanut allergic children reported by others [30, 49, 50], but in contrast with other studies, that reported a lower prevalence [24, 29]. Variations in reported prevalence of sIgE to peanut components may be linked to differences in study designs, geographical location and study populations.

We found, in line with other studies, a high rate of co-sensitization for Ara h 2 and Ara h 6 in peanut allergic children [24, 29, 31, 37, 51–53]. Ara h 2 and Ara h 6 are both seed storage proteins belonging to the 2S albumin family and they share a high amino acid sequence identity. However, mono-sensitizations to Ara h 6 occur in peanut allergic children. In our study we detected two peanut allergic child sensitized to Ara h 6, but not to Ara h 2. This enforces the added value of Ara h 6 sIgE determination in Ara h 2 negative children with a possible peanut allergy. This is similar to some other studies, who detected mono sensitizations of Ara h 6 in 1.2–18% of the peanut allergic children and adults [7, 24, 31, 50], but is in contrast with others that did not detect any Ara h 6 mono sensitization [33, 54].

Although we detected Ara h1 and Ara h3 in respectively 49% and 27% of the peanut allergic children, these storage proteins did not contribute to the diagnosis of peanut allergy in our population since no peanut-allergic patients were sensitized to Ara h 1 or Ara h 3 without co-sensitization to either Ara h 2 or Ara h 6.

Also other studies show a relatively high prevalence of sensitization to Ara h1 (between 40 and 94%) and Ara h 3 (between 23 and 77%), in peanut allergic children and adults, but only few mono-sensitizations to Ara h 1 or Ara h 3 are found in the peanut allergic population [24, 29, 33, 37, 50].

Sensitization to Ara h 8 was frequent in both the peanut allergic and peanut tolerant group, but not significantly different between both groups. The high prevalence of sIgE to the birch pollen homologue Ara h 8 is consistent with the high prevalence of birch pollen sensitization in our Dutch population comparable with other North European studies [19, 21, 50, 55].

Sensitization to the nsLTP component Ara h 9 was low in our population and did not contribute to the diagnostic accuracy peanut component analysis. This is in line with other reports from northern European populations [21, 49], but sensitization patterns in peanut allergic patients differ between geographical regions. In Southern Europe, LTP sensitization is more common and it was earlier shown that almost two third of peanut allergic patients from Spain and Greece are sensitized to the nsLTP protein Ara h 9 [19, 21]. In earlier studies, sensitization to multiple peanut storage proteins has been associated with a higher probability of clinically relevant peanut allergy [19, 37, 50, 55]. Our study shows similar results, 91% of peanut allergic children were sensitized to at least 2 storage proteins compared to 22% in the children with a negative peanut challenge. Mono-sensitization to storage proteins was less frequently found in the peanut allergic group.

There are some limitations of this study. In the peanut allergic group, children more often presented with allergic reactions to peanut in the history compared to the peanut tolerant group. This could have biased the results. However, another study showed no differences in specific IgE to peanut and major peanut allergens between challenge confirmed peanut allergic children with a clinical history to peanut compared to them with sensitization without earlier exposure to peanut [50].

Another limitation of this study was that 2 different peanut challenge test dosing schemes were used over time and between the two participating centers before 2014. This may have influenced the calculated sensitivity of the different sIgE tests and the associated negative predictive value. However, most patients with a negative peanut challenge succeeded in home introduction of the peanut. In 1 patient with a negative peanut challenge but increased Ara h 2 and Ara h 6 sIgE information regarding home introduction is missing due to loss to follow-up and 1 patient did not succeed to introduce peanut at home because of refusal.

## Conclusions

In conclusion, we showed that Ara h 2 and Ara h 6 sIgE determined using ISAC analyses are good predictors for peanut allergy in Dutch children. For both peanut components Ara h 2 and Ara h 6 we have now identified ISAC cut-off values relevant to predict a clinically relevant peanut allergy.

Ara h 2 sIgE determined by singleplex measurement shows a good correlation with Ara h 2 sIgE determined by multiplex ISAC microarray analysis. The simultaneous measurement of other peanut components sIgE using ISAC improves the diagnostic performance of this system and is a relevant addition.

Based on our findings we do not recommend Ara h 2 sIgE as a stand-alone measure of peanut sensitization in the patient that will be evaluated for possible peanut allergy. Clinicians should be aware that sIgE to other peanut components (Ara h 1, Ara h 3, Ara h 6, Ara h 8 and Ara h 9) may be relevant. A step wise approach is recommended in which, for our North-West European population, an additional Ara h 6 sIgE evaluation will increase the diagnostic accuracy in those individuals with negative Ara h 2 sIgE. As a result, allergic reactions in the home situation, associated with a small risk of peanut allergy in Ara h 2 sIgE negative patients, can thus be prevented.

## Data Availability

The datasets used and/or analysed during the current study are available from the corresponding author on reasonable request.

## Abbreviations

Ara h: Arachis hypogaea
AUC: Area under the curve
CRD: Component resolved diagnostics
FEIA: Fluorescence Enzyme Immunoassay
ISAC: Immuno Solid-Phase Allergen Chip
ISU: International standardized units
NPV: Negative predictive value
nsLTP: Non-specific lipid-transfer protein
OFC: Oral food challenge
PPV: Positive predictive value
PR-10: Pathogenesis-related protein family
ROC: Receiver operating curve
sIgE: Specific IgE
